# *Thermococcus sp*. 9°N DNA polymerase exhibits 3′-esterase activity that can be harnessed for DNA sequencing

**DOI:** 10.1038/s42003-019-0458-7

**Published:** 2019-06-20

**Authors:** Shiuan-Woei LinWu, Yu-Hsuan Tu, Ting-Yueh Tsai, Manuel Maestre-Reyna, Mu-Sen Liu, Wen-Jin Wu, Jyun-Yuan Huang, Hung-Wen Chi, Wei-Hsin Chang, Chung-Fan Chiou, Andrew H.-J. Wang, Johnsee Lee, Ming-Daw Tsai

**Affiliations:** 1 Personal Genomics, Inc., Zhubei, Hsinchu 30261 Taiwan; 2 Institute of Biological Chemistry, Academia Sinica, 128 Academia Road Sec. 2, Nankang, Taipei, 115 Taiwan; 3 Institute of Biochemical Sciences, National Taiwan University, Taipei, 106 Taiwan

**Keywords:** Next-generation sequencing, Enzyme mechanisms, X-ray crystallography, Biophysical methods

## Abstract

It was reported in 1995 that T7 and Taq DNA polymerases possess 3′-esterase activity, but without follow-up studies. Here we report that the 3′-esterase activity is intrinsic to the *Thermococcus sp*. 9°N DNA polymerase, and that it can be developed into a continuous method for DNA sequencing with dNTP analogs carrying a 3′-ester with a fluorophore. We first show that 3′-esterified dNTP can be incorporated into a template-primer DNA, and solve the crystal structures of the reaction intermediates and products. Then we show that the reaction can occur continuously, modulated by active site residues Tyr409 and Asp542. Finally, we use 5′-FAM-labeled primer and esterified dNTP with a dye to show that the reaction can proceed to ca. 450 base pairs, and that the intermediates of many individual steps can be identified. The results demonstrate the feasibility of a 3′-editing based DNA sequencing method that could find practical applications after further optimization.

## Abstract (web summary)

Shiuan-Woei LinWu et al. presents a DNA sequencing technology with high accuracy, using 9°N DNA polymerase. This study expands the current portfolio of enzymes equipped with 3′-esterase activity and shows the feasibility of a 3′-editing-based DNA sequencing.

## Introduction

As stated in a recent review commemorating 40 years of DNA sequencing, “DNA sequencing remains a young technology”^1^. Thus, despite the development of many highly efficient sequencing methods over the past four decades, there is room and need for further development and improvement^2,3^. At present, the most commonly used DNA sequencing approach is the cyclic reversible terminator approach of Illumina (one of the sequencing-by-synthesis methods), which is based on the incorporation of 3′-blocked terminators, followed by chemical reactions to remove the fluorophore from the nucleobase and restore the 3′-OH group^4^. This method, while highly popular, has its limitations in the need to pause for cleavage at each cycle, the residual fluorescent label at the nucleobase not cleaved completely in each cycle, and the relatively short read length^5,6^. Some of such limitations can be circumvented by two emerging single-molecule sequencing methods, from PacBio and Oxford Nanopore Technologies (ONT). The PacBio approach is also based on the conventional sequencing-by-synthesis^7^, whereas the ONT method relies on passing a DNA molecule through a nanoscale pore in a membrane^8^.

During the development of 3′-blocked terminators, it was reported that some of the 3′-derivatized dNTPs were not terminators, but instead were incorporated into DNA by DNA polymerases, such as T7 DNA polymerase lacking 3′–5′ exonuclease activity and HIV reverse transcriptase^9,10^. This finding suggested that the 3′-blocker was hydrolyzed during the process. Further studies indicated that T7 and *Taq* DNA polymerases possess a 3′-esterase activity^10^, which has limited the use of some 3′-blocked dNTPs as chain terminators. Potentially, this property can be employed to simplify the sequencing procedure by replacing the chemical methods to cleave the 3′-derivative^11^. However, the esterase activity of DNA polymerases remains to be characterized at the chemical and structural level, and its applicability in DNA sequencing remains to be demonstrated, particularly on a DNA polymerase that has already been shown to be suitable for DNA sequencing.

In this work, we demonstrate these properties using a 9°N DNA polymerase. We show that 3′-esterified dNTP can be incorporated into a template-primer DNA by using MS analysis and X-ray crystallography to validate reaction intermediates and products. Then we show that the reaction can occur continuously, and that its activity can be modulated by active site residues Tyr409 and Asp542. Finally, we use 5′-FAM-labeled primer and esterified dNTP with a dye to show that the reaction can proceed to long reads with reasonably good fidelity. The results demonstrate the 3′-esterase activity and its potential for further developing into a useful method for DNA sequencing.

## Results

### Incorporation of dNTP-3′-ester analogs by kinetic analysis

**Fig. 1:**
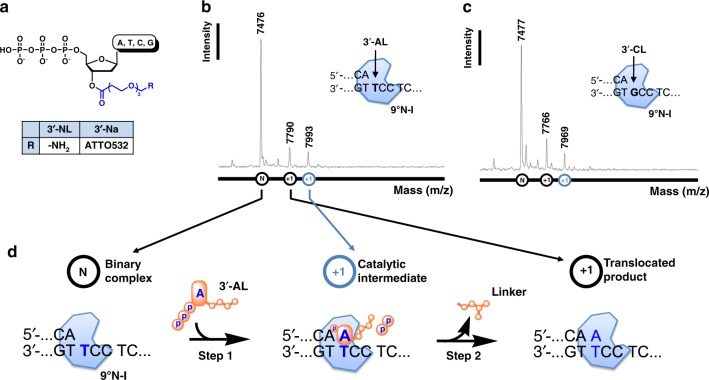
Demonstration of the 3′-esterase activity of 9°N-I DNA polymerase by MS. **a** Chemical structures of 3′-esterified dNTP nucleotides 3′-NL and 3′-Na used in this study. An ester-derived linker indicated in blue is connected to O3′ of dNTP, and the terminal group R represents −NH_2_ or ATTO532 fluorophore^13^. **b**, **c** Single-nucleotide primer extension assay employing 9°N-I with 3′-AL (**b**) and 3′-CL (**c**) in the presence of 2 mM MnCl_2_. The extension products were identified by MALDI-TOF/TOF mass spectrometry. **d** The proposed nucleotide incorporation and cleavage scheme of 9°N-I

The nucleotides dNTP used in this study were ester-modified at the 3′ position of the ribose, with a linker (3′-NL, where N stands for A, T, C, or G, and L stands for a linker) or a linker plus a dye (3′-Na, where “a” stands for a linker plus a dye) (Fig. 1a), and the enzyme used was an exonuclease-deficient variant (D141A and E143A) of the archaeal B family 9°N DNA polymerase^12^, with A485L mutation and named 9°N-I DNA polymerase (abbreviated as 9°N-I)^13^. The oligonucleotides used in this work are listed in Supplementary Table 1. We first used pre-steady-state kinetic assays^14,15^ to show that the substrates 3′-NL can be incorporated into DNA by 9°N-I in a single turnover event in the presence of Mg^2+^, though, as expected for substrate analogs, the *k*_pol_ values of 3′-NL were lower than those of dNTP by a factor of 6–15, while the *K*_d,app_ values were higher by a factor of 10–65 (Table 1).

**Table 1:** Summary of pre-steady-state kinetic data of dNTP and 3′-NL^a^

Nucleotide	*k*_pol_ (s^−1^)	*K*_d,app_ (μM)	*k*_pol_/*K*_d,app_	*k*_pol_/*K*_d,app_ (3′-AL/dATP)
*WT dNTP*				
dATP	43.9 ± 8.5	1.3 ± 0.4	36.4 ± 9.1	
dCTP	15.4 ± 2.7	5.2 ± 0.7	2.9 ± 0.2	
dTTP	19.8 ± 1.4	4.4 ± 1.3	4.7 ± 1.2	
dGTP	50.9 ± 8.7	2.7 ± 0.6	20 ± 7.5	
*3*′-*NL*				
3′-AL	2.8 ± 0.6	49 ± 5.6	0.06 ± 0.02	0.00165
3′-CL	2.6 ± 0.2	57.4 ± 17.3	0.05 ± 0.01	
3′-TL	2.5 ± 1	258.6 ± 8.3	0.01 ± 0.004	
3′-GL	6.4 ± 2.2	175.3 ± 31.4	0.04 ± 0.01	
*Y409A*				
dATP	1.6 ± 0.4	1 ± 0.4	1.6 ± 0.4	
3′-AL	0.05 ± 0.01	61 ± 7	0.001 ± 0.0001	0.00062
*D542E*				
dATP	2.7 ± 0.1	30.4 ± 3.2	0.1 ± 0.01	
3′-AL	0.03 ± 0.003	118.1 ± 19	0.0003 ± 0.00002	0.003

^a^The experiment was performed by adding 1 μM 9°N-I to the incorporation reaction in the presence of 2 mM MgSO_4_ (see the Methods section). The ± values stand for standard deviation based on three fitted lines for *k*_pol_ and *K*_d,app_ from three distinct aliquots of nucleotide samples (*n* = 3)

### Incorporation of dNTP-3′-ester analogs by MS analysis

We next used MALDI-TOF/TOF mass spectrometry (MS) to identify the product of single-nucleotide incorporation. As examples, Fig. 1b, c show the MS analysis for the incorporation of the 3′-esterified nucleotides 3′-AL (Fig. 1b) and 3′-CL (Fig. 1c) into DNA after the polymerase reaction in the presence of Mn^2+^. Based on the molecular weight of the extended primer and analysis of size standards (Supplementary Fig. 1), the linker was retained in the products from 3′-AL and 3′-CL, as indicated by +1 (blue) catalytic intermediate in addition to +1 (black) translocated product. However, the linker was absent from 3′-TL and 3′-GL incorporation (Supplementary Fig. 2). Similar results were observed in the presence of different divalent ions, Mg^2+^ and Ca^2+^ (Supplementary Figs. 3, 4). Although Ca^2+^ is usually inactive for DNA polymerases^16^, it appears to be accepted by the thermophilic KOD DNA polymerase^17^ and 9°N-I. As further described later, detection of intermediates with linker retained depends on the reaction conditions and the specific modified nucleotide used. The results here led us to describe the DNA incorporation reaction pathway of this 3′-esterified nucleotide catalyzed by 9°N-I in two steps, a nucleotidyl transferase reaction (step 1) to form a catalytic intermediate and an esterase reaction (step 2) to form a translocated product as shown in Fig. 1d.

### Structural evidence for the esterase activity

**Fig. 2:**
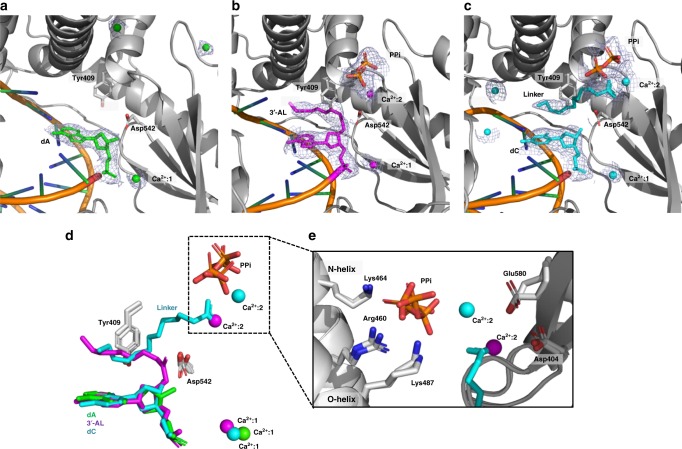
Structural evidence for the 3′-esterase activity of 9°N-I DNA polymerase. **a** The crystal structure of 9°N-I in complex with primer/template (P/T) duplex DNA with dATP incorporation. Simulated annealing 2Fo-Fc omit maps (light gray) centered on the translocated dAMP (green) and Ca^2+^ (green), and contoured at 1.0 σ are shown. The active-site residues Tyr409 and Asp542 are also indicated. **b** Same with **a**, except with 3′-AL incorporation, and centered on the incorporated monophosphate 3′-AL (magenta), PPi (orange) and Ca^2+^ (magenta), contoured at 0.8 σ. **c** Same with **a**, except with 3′-CL incorporation, centered on the translocated dCMP (cyan), cleaved 3′-linker moiety (cyan), PPi (orange) and Ca^2+^ (cyan), contoured at 1.0 σ. **d** Superimposed stick model of the incorporated nucleotides, PPi, residues Tyr409 and Asp542, and Ca^2+^ in the active site of 9°N-I from **a** to **c**. **e** Close-up view of the PPi interaction network from **d**

We further used structural analyses to examine the incorporation of 3′-NL into DNA. Before protein crystallization, the incorporation reaction was performed at 4 °C for 16 h with annealed primer-template DNA, dNTP or 3′-NL, and 9°N-I in the presence of Ca^2+^ (see the Methods section). Crystals grew in 2 weeks at room temperature and were frozen for X-ray diffraction. The crystallographic statistics for data collection and structure refinement are summarized in Table 2. Figure 2a shows the structure of the complex with the natural dAMP incorporated into DNA, thus the structure resembles that of the 9°N DNA polymerase:DNA binary complex^18^. The DNA with incorporated dAMP is translocated back to the original state, and a divalent ion (Ca^2+^:1) is near the phosphodiester bond. Similar results were obtained for dTTP, dCTP and dGTP (Supplementary Fig. 5). On the other hand, we observed the 3′-linker in the structure with 3′-AL (Fig. 2b), showing that the 3′-esterified nucleotide can also be directly incorporated into a DNA primer, and providing structural validation for the MS result in Fig. 1b. In this structure, the 3′-end extended linker of 3′-AL is located in a position between the adenine moiety of the incorporated nucleotide and the aromatic ring of Tyr409 above it, forming a so-called “sandwich effect” of the hydrophobic effect. This position normally resides the incoming nucleotide for incorporation^19^, as shown by the 3D alignment of the nucleotides in the active site (Supplementary Fig. 6a). In addition, the 3′ (−O−) site is located close to the side chain of Asp542. This site can also play a role in mediating divalent ions when the nucleotide enters the active-site region of 9°N DNA polymerase^19^. Interestingly, we also found that the pyrophosphate (PPi) product is located at the bottom of the finger subdomain of the active-site region, providing another evidence that the incorporation reaction of 3′-esterified dNTP did occur. Retention of PPi was also found in the structure with incorporated dGMP (Supplementary Fig. 5c).

**Table 2:** Summary of data collection and refinement statistics

	9°N-I/DNA/3′-AL	9°N-I/DNA/3′-CL	9°N-I/DNA/dA
*Data collection*			
Space group	I23	I23	P2_1_2_1_2_1_
Cell dimensions			
a, b, c (Å)	207.65, 207.65, 207.65	205.20, 205.20, 205.20	92.95, 106.77, 255.90
α, β, γ (°)	90.0, 90.0, 90.0	90.0, 90.0, 90.0	90.0, 90.0, 90.0
Resolution (Å)	48.9–3.3 (3.4–3.3)	40.2–3.2 (3.3–3.2)	46.3–2.8 (2.9–2.8)
R_merge_	0.0446 (0.47)	0.0238 (0.41)	0.0471 (0.34)
CC 1/2	0.991 (0.654)	0.999 (0.71)	0.994 (0.703)
Mean I/σ (I)	10.71 (1.59)	17.88 (1.96)	9.82 (2.41)
Completeness (%)	96 (98)	100 (100)	99 (99)
Redundancy	1.9 (1.9)	2.0 (2.0)	2.0 (2.0)
*Refinement*			
No. of reflections	21652 (2203)	23806 (2372)	63198 (6240)
R_work_/R_free_	0.2157/0.2778	0.2325/0.2902	0.2263/0.2758
No. of atoms	6207	6127	13390
Macromolecules	6143	6078	13170
Ligands	42	28	7
Solvent	22	21	213
B-factors	114.59	125.74	91.10
Macromolecules	114.62	125.64	91.53
Ligands	120.72	177.91	107.34
Solvent	92.20	86.19	64.02
Rms deviations			
Bond lengths (Å)	0.0020	0.0037	0.0033
Bond angles (°)	1.1842	1.2699	1.2222
*Ramachandran statistics*			
Favored (%)	91.79	90.33	97.48
Allowed (%)	8.21	9.67	2.52
Outliers (%)	0	0	0

	9°N-I/DNA/dT	9°N-I/DNA/dG	9°N-I/DNA/dC
*Data collection*			
Space group	P2_1_2_1_2_1_	I23	P2_1_2_1_2_1_
Cell dimensions			
a, b, c (Å)	92.09, 107.36, 255.67	208.08, 208.08, 208.08	92.76, 106.78, 258.09
α, β, γ (°)	90.0, 90.0, 90.0	90.0, 90.0, 90.0	90.0, 90.0, 90.0
Resolution (Å)	46.4–2.8 (2.9–2.8)	40.8–3.4 (3.5–3.4)	46.4–2.8 (2.9–2.8)
R_merge_	0.0743 (0.38)	0.0268 (0.48)	0.0388 (0.49)
CC 1/2	0.988 (0.632)	0.999 (0.585)	0.997 (0.498)
Mean I/σ (I)	4.60 (1.57)	14.20 (1.70)	13.78 (1.71)
Completeness (%)	99 (99)	100 (100)	99 (99)
Redundancy	2.0 (1.9)	2.0 (2.0)	2.0 (2.0)
*Refinement*			
No. of reflections	62970 (6160)	20707 (2046)	63594 (6276)
R_work_/R_free_	0.2328/0.2699	0.2539/0.2987	0.2162/0.2515
No. of atoms	13186	5922	13176
Macromolecules	13131	5878	12962
Ligands	7	14	63
Solvent	48	30	151
B-factors	63.02	157.75	84.26
Macromolecules	63.08	157.88	84.39
Ligands	86.26	185.51	99.99
Solvent	41.63	119.21	65.86
Rms deviations			
Bond lengths (Å)	0.0066	0.0021	0.0073
Bond angles (°)	1.3396	1.1693	1.3933
*Ramachandran statistics*			
Favored (%)	94.76	93.35	96.08
Allowed (%)	5.24	6.65	3.92
Outliers (%)	0	0	0

Statistics for the highest-resolution shells are shown in parentheses

We also analyzed the structure after the polymerase reaction with 3′-CL (Fig. 2c). In contrast to the structure from 3′-AL in Fig. 2b, the 3′-linker moiety of the 3′-CL breaks off the 3′-end of the ribose. The distal part of the 3′-CL-cleaved linker overlaps with the portion of the stacking linker that is retained on the 3′-AL structure in Fig. 2d, while the entire 3′-CL-cleaved linker shares similar binding site to that of the dATP bound to 9°N/DNA^19^ (Supplementary Fig. 6b). Furthermore, the proximal carboxylic acid moiety of this 3′-CL linker is close to PPi, mediated via Ca^2+^:2 that interacts with both. By aligning the 3′-AL and 3′-CL active-site structures (Fig. 2d), it was found that the pyrophosphates overlap well and interact directly with basic amino acids Arg460 and Lys464 of the N-helix and Lys487 of the O-helix, and a second divalent ion (Ca^2+^:2) (Fig. 2e).

### Incorporation of dNTP-3′-ester analogs can occur continuously

**Fig. 3:**
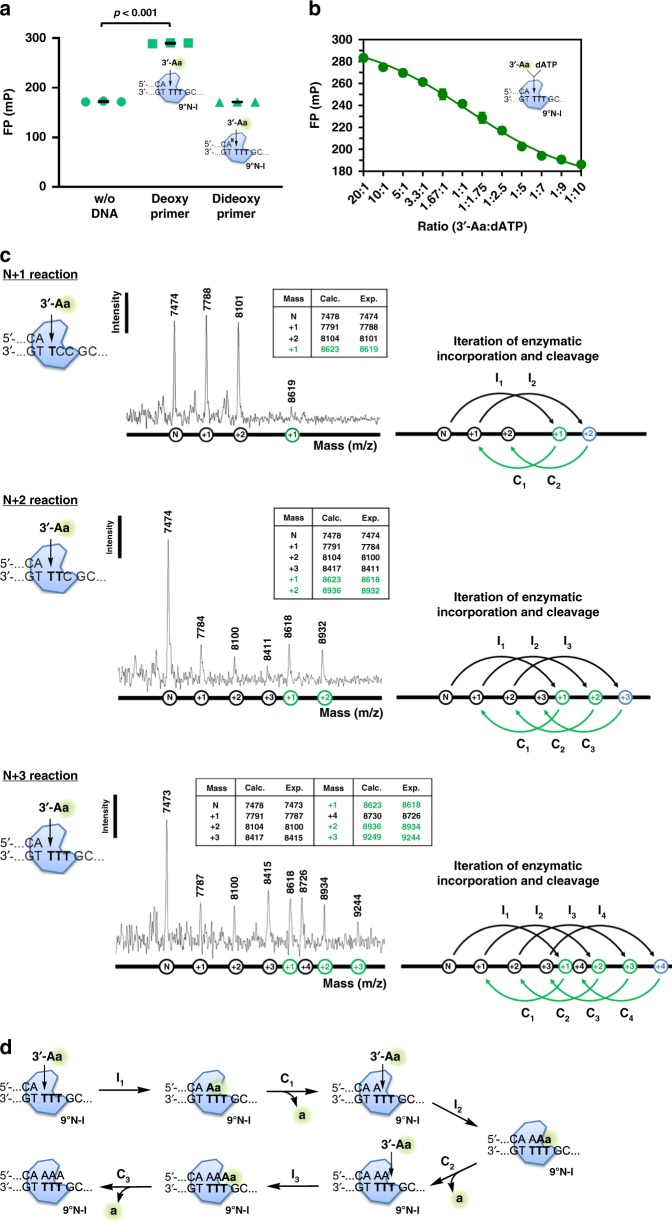
Demonstration of continuous incorporation of 3′-esterified nucleotides and influence by active-site residues. **a** Fluorescence polarization (FP) assay of 3′-Aa after incorporation into 2′-deoxy (5′-…CA) or 2′,3′-di-deoxy (5′-…CC^X^) primer by 9°N-I (*n* = 3) was performed as described in the Methods section. **b** Competition of FP activity with [3′-Aa]/[dATP] ratios from 20:1 to 1:10 (*n* = 3). **c** Iteration flow of 3′-Aa incorporation and cleavage steps by 9°N-I and the sequential dT_3_ homopolymeric templates (+1, +2 and + 3) using MALDI-TOF/TOF MS as described in Fig. 1b. **d** Scheme for continuous extension of 3′-Aa by 9°N-I. The iterative steps consisting of the incorporation (I_1_, I_2_, and I_3_) of 3′-Aa and the cleavage (C_1_, C_2_, and C_3_) of 3′-modified fluorescent moiety “a” for extension of the dT_3_-based template

For the esterase activity to be applicable to DNA sequencing, it is necessary to show that the incorporation reaction of the 3′-esterified nucleotides into DNA can be continued, and also to develop an analytical method, particularly one with fluorescence-based detection, to monitor the continuous reaction. For these purposes, we designed a template strand with three consecutive thymidines (dT_3_), and first performed the reaction with 3′-end deoxy or di-deoxy primers in the presence of Mn^2+^, with the 3′-Aa substrate containing the fluorescent dye ATTO532 (Fig. 1a). As shown in Fig. 3a, since no chemical reaction is expected for the primer with a di-deoxy group, it shows the same basal fluorescence polarization (right dot plots) as that of the control without DNA (left dot plots), while the deoxy primer was able to gain fluorescence (middle dot plots), supporting that the 3′-linker-dye of 3′-Aa is retained as an intermediate on the DNA. Next, we performed dose-competitive reactions of dATP on 3′-Aa. The results showed that as the proportion of dATP increased, the FP intensity decreased correspondingly (Fig. 3b).

We again used MS to analyze the reaction products. The template strands for performing N + 1 (5′-…CGCC**T**…), N + 2 (5′-…CGC**TT**…), and N + 3 (5′-…CG**TTT**…) reactions led to two sets of extended primers with the expected mass increase, one with the extension of dAMP (+1, +2, and +3; black circles) and the other with the linker-dye moiety retained at the 3′ end (+1, +2, and +3, green circles) (Fig. 3c). Note that an extra mismatched incorporation occurred in each reaction as indicated in blue circles, for which the intermediates were not detected. These results confirmed that the sequential iteration of enzymatic incorporations (I_1_, I_2_, I_3_…) and cleavages (C_1_, C_2_, C_3_…) of 3′-Aa upon the primer strands via 9°N-I occurred (Fig. 3c), indicating that the 3′-esterified nucleotides can be incorporated into DNA in a continuous manner through the stepwise flows of nucleotide incorporation and cleavage (Fig. 3d).

### The 3′-esterase activity is intrinsic to 9°N-I

**Fig. 4:**
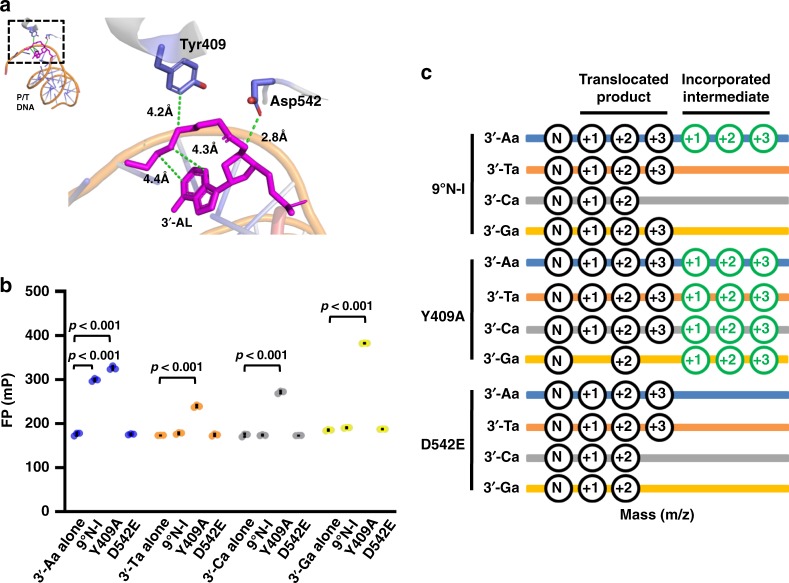
The esterase activity is modulated by active site residues. **a** Involvement of Tyr409 and Asp542 at the active site of 9°N-I in the cleavage of 3′-esterified nucleotide. The distance between 3′-esterified moiety of 3′-AL (colored in magenta) and Tyr409 or Asp542 (colored in blue) are shown. **b** FP activity of 3′-Na (including 3′-Aa, 3′-Ta, 3′-Ca, and 3′-Ga) after incorporation into the primer by 9°N-I and its mutants Y409A and D542E (*n* = 3). The primer extension was performed with dN_3_-based templates. **c** MALDI-TOF/TOF MS profiles of the incorporated intermediates of 3′-Na catalyzed by both Y409A and D542E. After primer extension, the detected ester intermediates and translocated products (+1, +2, and +3) were colored in green and black, respectively. Details are described in Supplementary Figs. 8–10

For the 3′-esterase activity to be useful for development into a novel strategy for DNA sequencing, it is also important to show that this activity is an intrinsic property of the enzyme (i.e., it is mediated by active-site residues), and that its activity and specificity can be further optimized by protein engineering. Based on the structural analyses described above, we further investigated whether the active-site residues Tyr409 and Asp542 (Fig. 4a) are involved in the 3′-esterase activity. We performed saturation mutations at these sites, and screened each mutant for FP fluorescence after incorporating each of the four ATTO532 dye nucleotides 3′-Na (3′-Aa, 3′-Ta, 3′-Ca, and 3′-Ga) into DNA of dN_3_-based templates (dT_3_, dA_3_, dG_3_, and dC_3_). It was observed that Y409A displayed the greatest change relative to wild-type 9°N-I and retained FP fluorescence for all four 3′ dye-modified nucleotides, whereas none of the Asp542 mutants retained FP fluorescence for any of the substrates (Supplementary Fig. 7). Figure 4b shows the comparison between the wild-type 9°N-I, which retained fluorescence for only 3′-Aa, and the Y409A (retaining fluorescence for all four nucleotides) and D542E (no fluorescence for any nucleotide) mutants. It is important to emphasize here that the lack of observed fluorescence does not mean lack of incorporation; it should indicate the lack of retention of the intermediate. To verify this point, mass spectrometry was performed to analyze the incorporation with 3′-Na by 9°N-I, Y409A and D542E, which are shown in full in Supplementary Figs. 8–10. As summarized schematically in Fig. 4c, all three enzyme variants could incorporate all four 3′-Na nucleotides, but the WT 9°N-I only retained intermediates of 3′-Aa, and Y409A retained the intermediates of all four nucleotides, while D542E retained none, which are in full agreement with the FP results in Fig. 4b.

While detection of the ester intermediate (after incorporation of 3′-NL or 3′-Na into DNA) suggests that the rate of ester hydrolysis is slower (relative to the cases where the intermediate is undetectable), it could not be measured quantitatively. Nonetheless, we determined the (*k*_pol_/*K*_d,app_) values for the two mutants for dATP and 3′-AL. As shown in Table 1, the catalytic activities of both mutants decrease relative to WT for both dATP and 3′-AL substrates, but the (*k*_pol_/*K*_d,app_)_3__′__-AL_/(*k*_pol_/*K*_d,app_)_dATP_ ratio of Y409A is lower than WT by 3x and that of D542E is higher than WT by 2× . This result is consistent with the enhanced detection of the ester intermediate for Y409A (relative to WT) and the lack of detection for D542E.

### Long read and low error rate

**Fig. 5:**
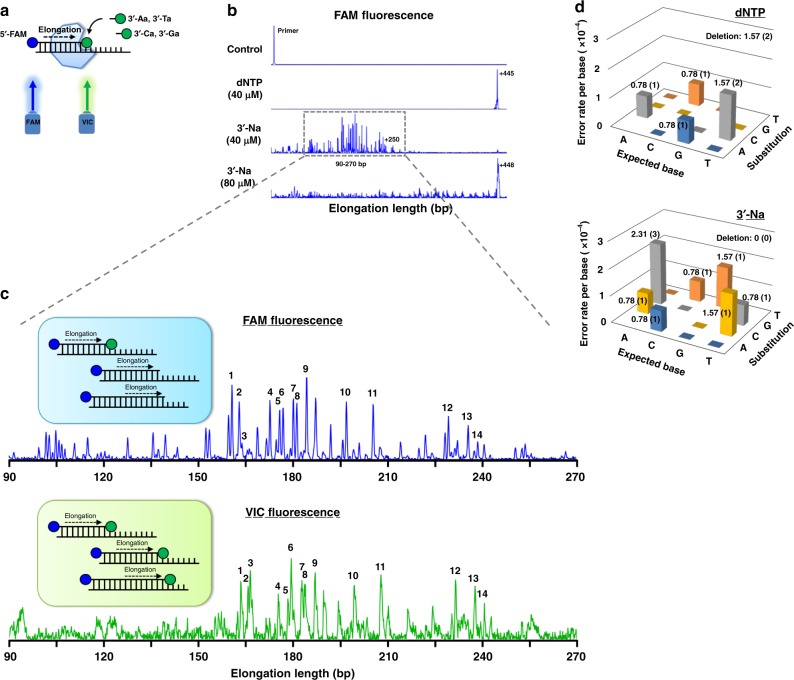
Demonstration of the feasibility of DNA sequencing by synthesis based on the 3′-esterase activity. **a** Monitoring of DNA elongation by capillary electrophoresis with 5′-FAM-labeled primer (50 bases) annealed to the corresponding template region linking to a 400 base-long M13 ssDNA (Oligo_T#19). The extended oligonucleotides are visualized using FAM fluorescence (blue) at 5′ position and VIC fluorescence (green) for the incorporated ATTO532 fluorophore of 3′-Na at 3′ position. **b** Capillary electropherograms for the extension of 10 nM DNA primer by 40 μM dNTP and 40 and 80 μM 3′-Na at 60 °C for 1 h. Multiple reaction intermediates were observed for the reaction with 40 μM 3′-Na. **c** Alignment of FAM and VIC fluorescence-based capillary electropherograms in the extension region of 90–270 bp from panel **b**. As illustrated, the FAM fluorescence peaks (upper panel) include both the translocated products (5′-FAM-labeled oligonucleotides) and the catalytic intermediates (both 5′-FAM- and 3′-ATTO532-labeled oligonucleotides), while the VIC fluorescence peaks (lower panel) represent only the catalytic intermediates. Numbers 1–14 (red) denote the comparable signals collected from FAM and VIC fluorescence, with detailed comparison shown in Supplementary Table 2. **d** Profiles of the error rates (including substitution and deletion errors) of 9°N-I toward dNTP and 3′-Na, summed individually. Error rate per base (x10^−4^) is indicated in each column, and the number of mutation bases is shown in parentheses

Finally, we show that the continuous incorporation of 3′-Na can be used for a long read, by fluorescence, and with a low error rate. We took a single-stranded DNA fragment (450 bases in length) of the M13 phage genome as a template for the DNA synthesis reaction (Fig. 5a). By detecting the fluorescence of the 5′-FAM-labeled primer (blue peaks), it was found that the incorporation of 3′-Na can extend to the end of the template similar to the regular dNTPs, though a 2x concentration is required for the former (Fig. 5b). Furthermore, the fluorescence arising from FAM showed multiple intermediate primers. We then compared the 5′-FAM-labeled primers within the extension range of specific primers with the ATTO532-labeled intermediates, which could be illuminated similarly like VIC fluorescence (green peaks). The positions of the fluorescent signals in the FAM- and VIC-based electropherograms were found to be highly consistent, as indicated by the peaks numbered in black (Fig. 5c; Supplementary Table 2). Additional sets of experiments with multiple time points were also performed for 9°N-I (Supplementary Fig. 11a) and another archaeal B family polymerase KOD^13^ (Supplementary Fig. 11b). These results support that the intermediates in a long sequencing can be detected by fluorescence, though not all steps can be resolved at the current stage. Even though the same dye was used for all four nucleotides in this study, different fluorophores (e.g., four different dye nucleotides) can be adopted for base calling in the future.

Finally, the products from primer extension with dNTP and 3′-Na were subcloned and subjected to Sanger sequencing with thirty clones each to evaluate the catalytic error rates of 9°N-I (Supplementary Fig. 12 and Methods). As shown in Fig. 5d, the error rates of 3′-Na (8.6 × 10^−4^) and dNTP (5.5 × 10^−4^) from 12,720 bases sequenced are both low and comparable with each other.

## Discussion

We report to our knowledge a new sequencing chemistry by designing 3′-esterified nucleotides to work with the 9°N-I DNA polymerase, and demonstrate its feasibility for DNA sequencing by synthesis in a continuous manner with a low error rate. This sequencing chemistry involves not only an incorporation step but also a “lagging period” due to processing of the 3′-blocking group of the nucleotide by the polymerase. Our results further show that it is possible to modulate the esterase activity, thus the detectability of the fluorescent intermediate, by changing experimental conditions and engineering active-site residues. It is highly promising that, after further optimization and integration into a fluorescent sensing platform, the 3′-esterified nucleotide-based sequencing chemistry could find practical applications as an advantageous NGS sequencing method, such as single-molecule real-time DNA sequencing, by attaching different dyes to the four 3′-Na nucleotides.

**Fig. 6:**
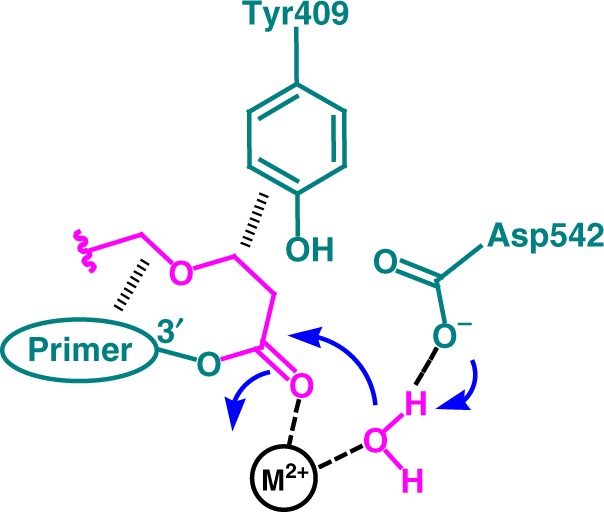
A proposed mechanism of the esterase activity of 9°N-I on a 3′-esterified nucleotide. Hydrolysis of the 3′-ester bond of the nucleotide, after incorporation into the primer, may be mediated by the side chain of Asp542 in conjunction with one of the active site divalent metal ions

Based on the results of MS analysis and pre-steady-state kinetics of Y409A and D542E mutants, we propose a hypothesis that the esterase activity of 9°N-I is mediated by Asp542 as a general base, in cooperation with one of the active-site metal ions, along with Tyr409 that stabilizes the linker (Fig. 6). Asp542 is known to act as a mediator of divalent ions that participate in the incorporation^16,19,20^. The D542E mutation is a conservative change, but is sufficient to enhance the esterase activity (relative to the incorporation activity) presumably because the Glu542 side chain is closer to the 3′-ester bond than the Asp542 side chain. For Tyr409, it is speculated that removal of its side chain in Y409A may reduce the hydrophobic interaction with the connecting part at the 3′-end of the linker and increase its flexibility, thus perturbing or destabilizing its contact with the side chain of Asp542 and leading to reduced esterase activity.

## Methods

### Preparation of nucleotide, polymerase, and DNA

The 3′-esterified nucleotides were designed and synthesized by the chemical synthesis laboratory (Personal Genomics, Inc.)^13^, and the structure information was identified using NMR and MS methodologies. The purities of all synthetic dNTP analogs are >99%. 9°N-I, an exonuclease-deficient variant (D141A and E143A) with A485L mutation^13^ derived from the archaeal B family 9°N DNA polymerase (9°N)^12^, and site-directed mutants of Tyr409 and Asp542 were constructed, expressed, purified, and stored as previously described^12,13,18,20^. KOD^exo−^, an exonuclease-deficient variant (D141A and E143A) with A485L mutation derived from *Thermococcus kodakaraensis* (KOD) DNA polymerase, was also prepared as described^13,18^. All oligonucleotides were synthesized, and the sequences of the primer-templates (P/Ts) used in this study are shown in Supplementary Table 1. For DNA elongation and pre-steady-state kinetic study, the primer was 5′-labeled with fluorescent dye 6-carboxyfluorescein (6-FAM) as described^21^.

### Pre-steady-state kinetic study

The rate of incorporation of the correct base onto the 50/67 nt duplex DNA was determined in a reaction. The reaction was initiated by combining the solution containing the enzyme and DNA substrate with the second solution containing Mg^2+^/dNTP^21^. The reaction was quenched with 0.1 M EDTA, pH 8.0. A rapid quench instrument (KinTek Instrument Corp., State College, PA) was used for the reaction time ranging from 100 ms to 60 s at varying concentrations of nucleotides^14,15^. DNA substrates used in the extension assays are listed in Supplementary Table 1. DNA annealing was performed with 60 nM 5′-FAM-primer (50 nt) and 120 nM template (67 nt) at 80 °C for 5 min, and slowly cooled to 25 °C for 10 min in 1x reaction buffer containing 10 mM (NH_4_)_2_SO_4_, 10 mM KCl, and 5 mM MgSO_4_ in 20 mM Tris-HCl (pH 7.5) with 0.1% Triton^®^ X-100. The final concentration of the annealed DNA in the incorporation reaction was 30 nM. The incorporation of regular (0.1–20 μM) and 3′-esterified (10–300 μM) nucleotides was initialized by adding the enzyme-DNA solution containing 1 μM 9°N-I and quenched by adding 0.1 M EDTA, pH 8.0. Kinetic measurements of incorporation were accomplished by KinTek RQF-3 quench-flow instrument at 60 °C for 0.1, 0.5, 1, 5, 10, 20, 30, or 60 s in 1x reaction buffer. The fluorescence and size of extended products were determined by capillary electrophoresis on an ABI 3500 genetic analyzer (Applied Biosystems) using POP-7 polymer and 36-cm length capillary^22^. The data were analyzed using GeneMapper Software 5 (Applied Biosystems) with specific detection parameters and displayed as a linear-log plot of product formation versus compound concentration.

### MALDI-TOF/TOF MS analysis

Mass measurement of DNA after nucleotide incorporation was described^23^ and performed with Bruker AutoFlex III smartbeam TOF/TOF 200 system (Bruker Daltonics, MA). DNA substrates used in the extension assays are listed in Supplementary Table 1. The primer (0.3 μM) was pre-annealed with the template at a 1:1 ratio in 1x reaction buffer (10 mM KCl and 2 mM divalent ions in 10 mM Tris-HCl, pH 7.5) by heating to 95 °C for 1 min, 55 °C for 2 min, and then cooling to 30 °C for 2 min. 9°N-I (0.4 μM) was added into the P/T duplex DNA and incubated on ice for 30 min. The 3′-esterified compound (40 μM) was then added into the polymerase reactions on ice and incubated at 60 °C for 30–60 min. The reaction was quenched with 2 μL of acetonitrile to stop the reaction. Before MS analysis, the reaction product was cleaned up with Micro Bio-Spin™ 30 column (Bio-Rad, CA). Before MS analysis, the sample was prepared by mixing 0.5 μL of the desalted products and 0.5 -μL matrix 3-HPA (3-hydropicolinic acid) in acetonitrile. The sample was dried and then analyzed^23^. The positive ion mode was used to collect all spectra.

### Crystallization and structure determination

Before crystallization, the 9°N-I was mixed in an equimolar ratio with a freshly annealed P/T to give a final protein concentration of 66–111 μM. The normal dNTPs or 3′-NL (3′-AL and 3′-CL) were then added to a final concentration of 1.11 mM. A solution of 10 mM CaCl_2_, 10% (v/v) glycerol, 25–30% (v/v) 2-methyl-2,4-pentanediol (MPD), and 100 mM sodium acetate (pH 4.6) was mixed with an equal volume of the protein complex. Square cube-shaped crystals grew within 2 weeks at room temperature, and had a typical dimension of ~100 × 100 × 100 μm. Crystals were transferred to a cryoprotectant solution where the concentration of MPD was 30% v/v prior to freezing in liquid nitrogen. The data were collected at 110 K using the synchrotron radiation sources at beamline 15A1 of Taiwan Photon Source (TPS), National Synchrotron Radiation Research Center (NSRRC, Hsinchu, Taiwan). The data were processed using the HKL2000 program suite^24^. The structures were solved by molecular replacement with a previously determined 9°N/DNA binary structure (4K8X)^18^, and refined using REFMAC5^25^. The dNTP and 3′-NL structures were built using the program COOT^26^. The data collection and structure refinement statistics are summarized in Table 2. All figures were prepared with PyMOL (Schrodinger, LLC)^27^.

### Fluorescence polarization assay

The fluorescence polarization (FP) experiment was performed and calculated based on the established method^28^. DNA substrates used in the extension assays are listed in Supplementary Table 1. The primer (10 μM) was pre-annealed with the template at a 1:1 ratio in 1x reaction buffer [10 mM (NH_4_)_2_SO_4_, 10 mM KCl, and 2 mM MnCl_2_ in 20 mM Tris-HCl, pH 7.5] before mixing with 3′-Na and 9°N-I. The solution was heated to 95 °C for 1 min, 55 °C for 2 min, and then cooled to 30 °C for 2 min. The 9°N-I (1 μM) was added into the P/T duplex DNA and incubated on ice for 30 min. The 3′-Na (0.5 μM) was added into polymerase reactions on ice and incubated at 60 °C for 60 min. Before fluorescence measurement, the reaction was quenched with 0.5 M EDTA (1.4 μL). For the incorporation assay, the deoxy P/T (Oligo_P#1 & Oligo_T#15) and di-deoxy P/T (Oligo_P#4 & Oligo_T#13) duplex DNA were used for the extension reaction. For the competition assay^29^, the P/T duplex DNA (Oligo_P#1 & Oligo_T#15) was used for the incorporation of the different ratios of [3′-Aa] to [dATP] (20:1 to 1:10) starting from 0.5 μM 3′-Aa.

#### FP Measurement

After the primer extension reaction, the reaction products were transferred to a Greiner Bio-One 96-well microplate for FP measurement on a Paradigm Multi-Mode Microplate Detection Platform under excitation at 535 nm and emission at 585 nm (Beckman Coulter, Brea, CA). Fluorescence polarization value was calculated using the formula:

$$P = \left[ {Ivv-Ivh} \right]/\left[ {Ivv + Ivh} \right]$$

where *Ivv* is the emission intensity measured when the excitation and emission polarizers are parallel, and *Ivh* is the emission intensity measured when the emission and excitation polarizers are oriented perpendicular to each other. The degree of polarization is expressed by the unit mP, or a 0.001 ratio between (*Ivv* − *Ivh*) and (*Ivv* + *Ivh*)^28^.

### DNA elongation using 3′-Na

The reaction was initiated by combining the solution containing the enzyme and DNA substrate with the second solution containing Mg^2+^/dNTP^21^. DNA substrates used in the extension assays are listed in Supplementary Table 1. DNA annealing was performed with 10 nM 5′-FAM-primer (50 bases, Oligo_P#5) and 60 nM M13 ssDNA template (450 bases, Oligo_T#19) at 80 °C for 5 min, and slowly cooled to 25 °C for 10 min in 1x reaction buffer containing 10 mM (NH_4_)_2_SO_4_, 10 mM KCl, and 5 mM MgSO_4_ in 20 mM Tris-HCl (pH 7.5) with 0.1% Triton^®^ X-100_._ The incorporation of 40 μM dNTP and 40–80 μM 3′-Na was initialized by adding 1 μM 9°N-I or KOD^exo−^ for 1 h (or 5, 15, 30, and 60 min in time-dependent studies) at 60 °C and quenched by adding 0.1 M EDTA, pH 8.0. The fluorescence and size of extended products were determined by capillary electrophoresis on an ABI 3500 genetic analyzer (Applied Biosystem) using POP-7 polymer and 36-cm length capillary^22^. The data were analyzed using GeneMapper Software 5 with specific detection parameters and displayed as a linear-log plot of product formation vs. compound concentration.

*Amplification and sequence analysis of extension products*. Extension reactions for fidelity analysis were performed as previous described^29,30^. Briefly, extending a P/T duplex DNA was performed in a 20 -μL reaction volume that contains 1 pmol of M13 template (Oligo_T#20) and 1 pmol of primer (Oligo_P#6) (Supplementary Fig. 12). The primer and template were annealed in 1x ThermoPol buffer by heating for 5 min at 85 °C and slowly cooling to 25 °C. The P/T duplex DNA contains a T–T mismatch, which produces a T to A transversion in the cDNA strand. The transversion represents a watermark to ensure that the sequenced DNA was produced by extension. The extension product was purified by spin-column method (Qiagen PCR clean-up kit). The purified extension products were amplified by using Phusion High-Fidelity DNA Polymerase (Thermo Fisher Scientific, Waltham, MA) with Oligo_P#7 and Oligo_P#8 according to the manufacturer’s instructions. PCR program: 98 °C for 3 min, then 35 cycles of: 98 °C for 30 s, 55 °C for 15 s, and 72 °C for 30 s. The amplified products were A-tailed by using 1U Taq polymerase (NEB) with 200 μM dATP in reaction buffer for 30 min at 37 °C. The tailing products were purified by Qiagen PCR clean-up kit, ligated into T&A vector (Yeastern Biotech, Taipei, Taiwan) following the manufacturer’s protocol. The ligated product was transformed into *Escherichia coli* DH5α. Individual colonies (30 clones each for dNTP or 3′-Na experiment) were grown in liquid media and submitted to DNA sequencing service (MB Biotech, Taipei, Taiwan). DNA sequences were aligned with M13 template (Oligo_T#20) and analyzed using BioEdit 7.0 (IIbis Therapeutics, Carlsbad, CA). Sequences lacking the T to A watermark were discarded as they were generated from the starting DNA template rather than replicated material.

### Statistical and reproducibility

In all figures, error bars represent s.d. of the mean, unless otherwise noted. To determine product and intermediate formation from 9°N-I and mutants on different substrates using MALDI-TOF/TOF MS, the product of three repeats (*n* = 3) of same sample were measured to reveal similar mass spectra of the enzyme product. FP assay was performed using purified 9°N-I and mutant enzyme with three repeats (*n* = 3) of same sample in every experiment (shown as mean ± s.d.), and two experiments were performed for individual assay. For the comparison between the groups, a paired *t*-test statistics analysis was performed with *p*-value (<0.001) indicating significant difference. DNA elongation was measured twice of same sample with similar results. Pre-steady-state kinetic study was measured from purified proteins and measurement were taken with three repeats (*n* = 3) of fitting lines for kinetics that generated individual *k*_pol_ and *K*_d,app_ values from different aliquot of the nucleotide substrates.

### Reporting summary

Further information on research design is available in the Nature Research Reporting Summary linked to this article.

## Supplementary information

Supplementary Information

Description of Supplementary Data

Reporting Summary

Supplementary Data 1

## Data Availability

The solved structures were deposited in the Protein Data Bank with the following accession codes: 9°N-I/DNA/dA (6IS7), 9°N-I/DNA/3′-AL (6ISH), 9°N-I/DNA/3′-CL (6ISI), 9°N-I/DNA/dT (6ISF) and 9°N-I/DNA/dG (6ISG). The source data underlying Figs. 3a, 3b, 4b, 5d and Supplementary Fig. 7 are presented in Supplementary Data 1.
