# Supplementary material for: Thermococcus sp. 9°N DNA polymerase exhibits 3′-esterase activity that can be harnessed for DNA sequencing
Source: Commun Biol. 2019 Jun 20;2:224. doi: 10.1038/s42003-019-0458-7 (PMC6586783; doi:10.1038/s42003-019-0458-7)
Supplement: Supplementary file 1 — Supplementary Information [file 42003_2019_458_MOESM1_ESM.pdf]

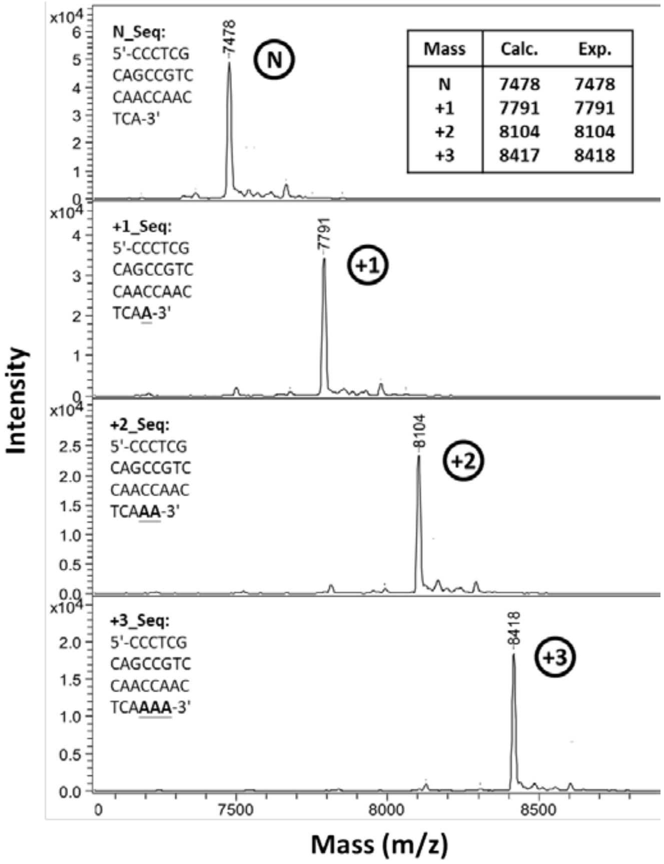

Supplementary Fig. 1. Size standards of +1, +2 and +3 translocated products from the commercially synthesized oligonucleotides, analyzed by MALDI-TOF/TOF mass spectrometry.

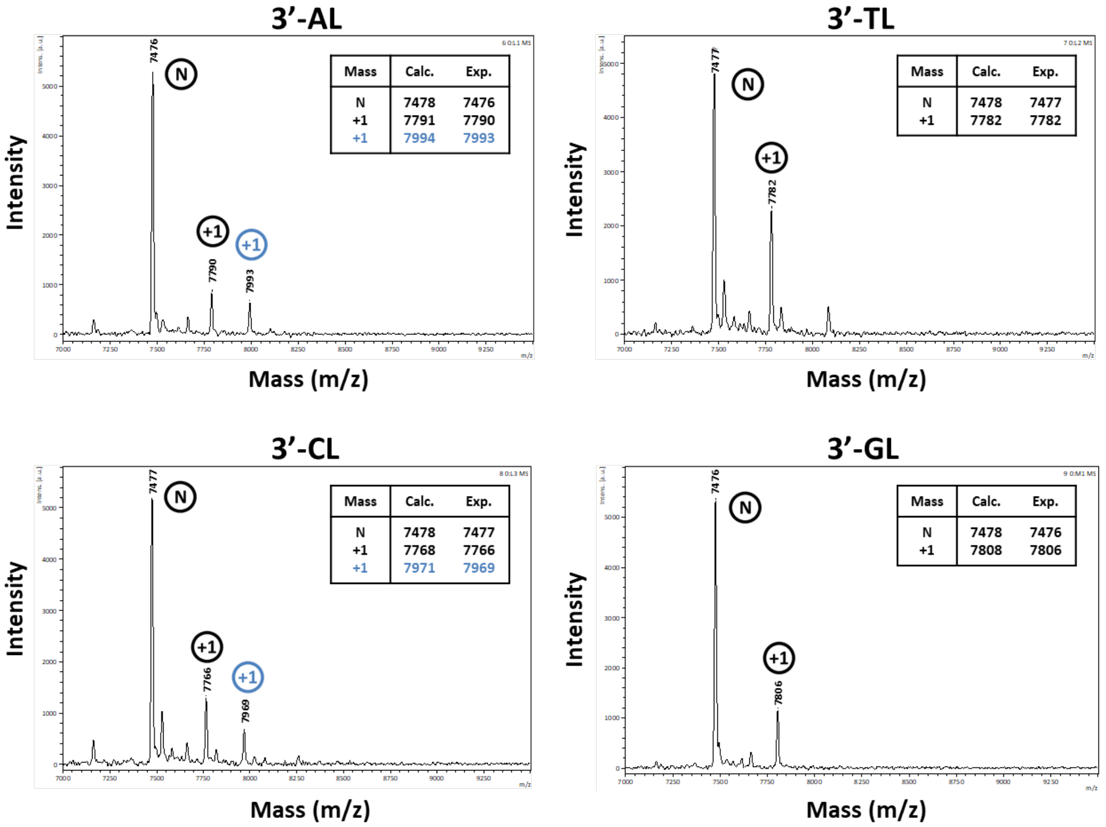

Supplementary Fig. 2. Single nucleotide incorporation of 3’-NL into DNA in the presence of Mn^{2+}. Extension of primer (Oligo_P#1, 0.3 μM) annealing to the respective template (Oligo_T#1-4, 0.3 μM) with 10 μM 3’-NL (3’-AL, 3’-TL, 3’-CL and 3’-GL) using 0.4 μM 9°N-I in the presence of 2 mM $MnCl_2$ was performed at 60 ˚C for 30 min and analyzed by MALDI-TOF/TOF mass spectrometry. The translocated products of one base (+1) are colored in black whereas the catalytic intermediates (+1) appeared in 3’-AL and 3’-CL are colored in blue. The experimental (Exp.) and calculated (Calc.) mass-to-charge ratio (m/z) of the extension peaks with the charge (z) of 1 are listed.

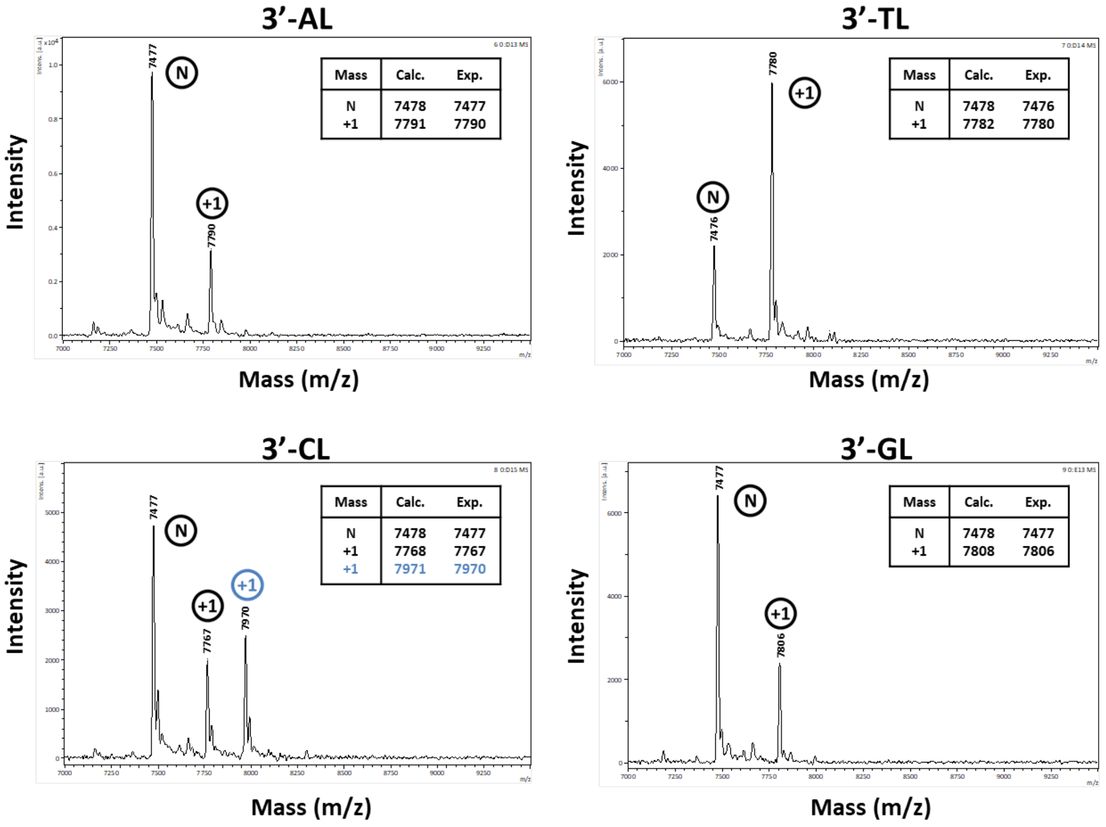

Supplementary Fig. 3. Single nucleotide incorporation of 3'-NL into DNA in the presence of Mg^{2+}. Same as **Supplementary Fig. 2**, except that 2 mM $MgSO_4$ was used.

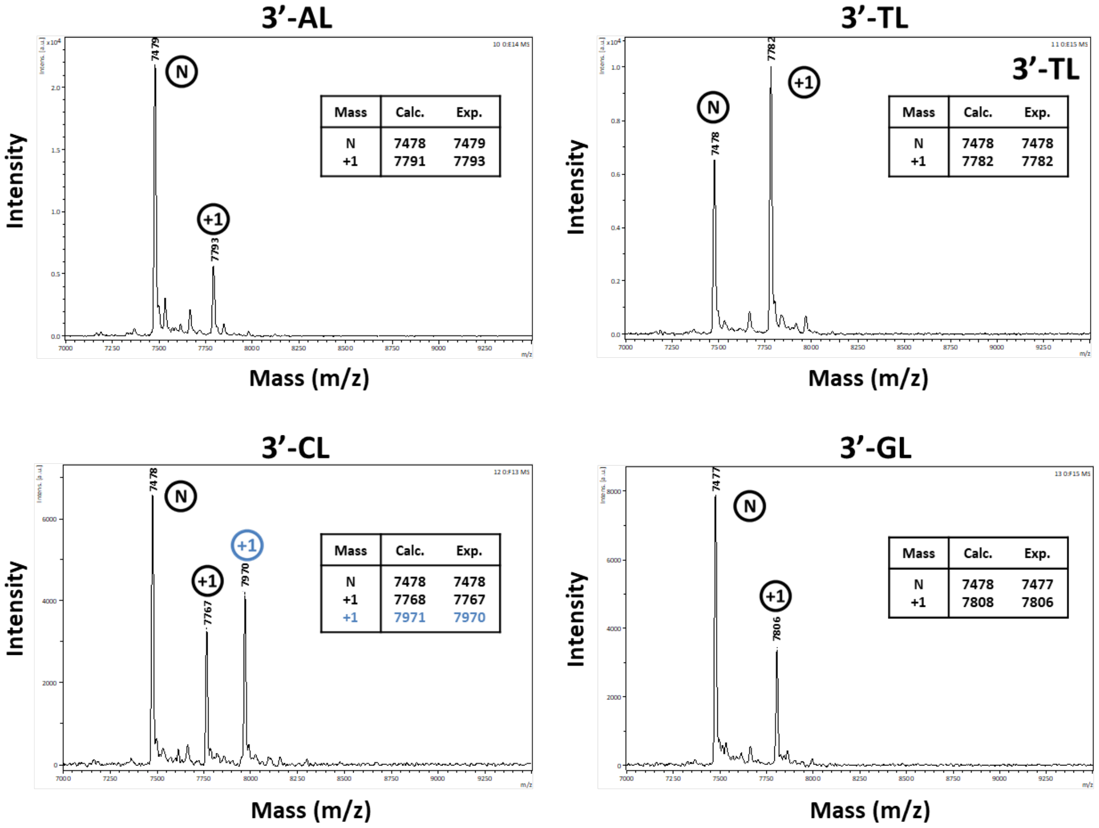

Supplementary Fig. 4. Single nucleotide incorporation of 3'-NL into DNA in the presence of Ca^{2+}. Same as **Supplementary Fig. 2**, except that 2 mM $CaCl_2$ was used.

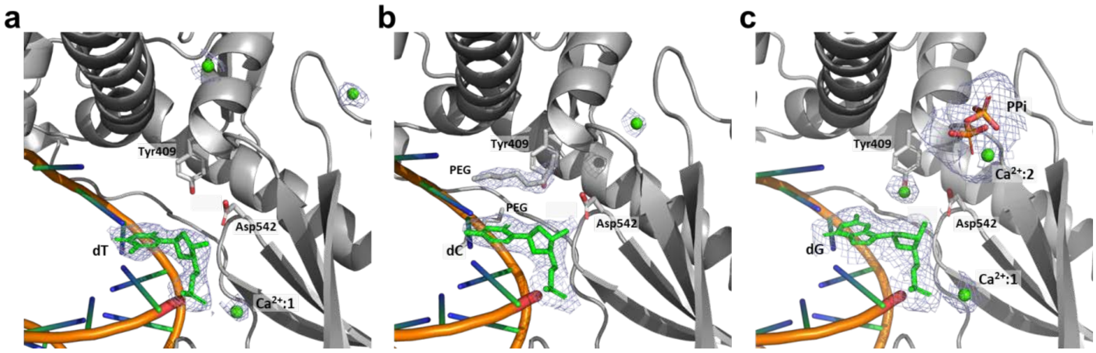

Supplementary Fig. 5. Structures of dNTP incorporation into DNA. Crystal structures of 9°N-I in complex with template/primer DNA with dTMP, dCMP and dGMP were determined after the incorporation reaction at 4 °C for 16 hours (**Table 2**). The incorporated dNMP and divalent atoms (Ca^{2+}) are colored in green. Simulated annealing 2Fo-Fc omit maps centered on the incorporated nucleotides, PPi and Ca^{2+}, and contoured at 1.0 σ are shown in light gray. The DNA with incorporated dNMP is translocated back to the original state. The divalent ion Ca^{2+}:1 is near the phosphodiester bond of dT (**a**) and dG (**c**), and the second divalent ion Ca^{2+}:2 interacts with PPi in dG (**c**). In addition, PEG molecules exist on top of and nearby dC (**b**) due to its specific crystallization condition with PEG300 similar to the previous study[19]. The solved structures were deposited in the Protein Data Bank with the accession codes: 9°N-I/DNA/dT (6ISF) and 9°N-I/DNA/dG (6ISG), except that the complex with dC was not deposited due to the presence of a free dCTP in one of the 9°N-I monomers.

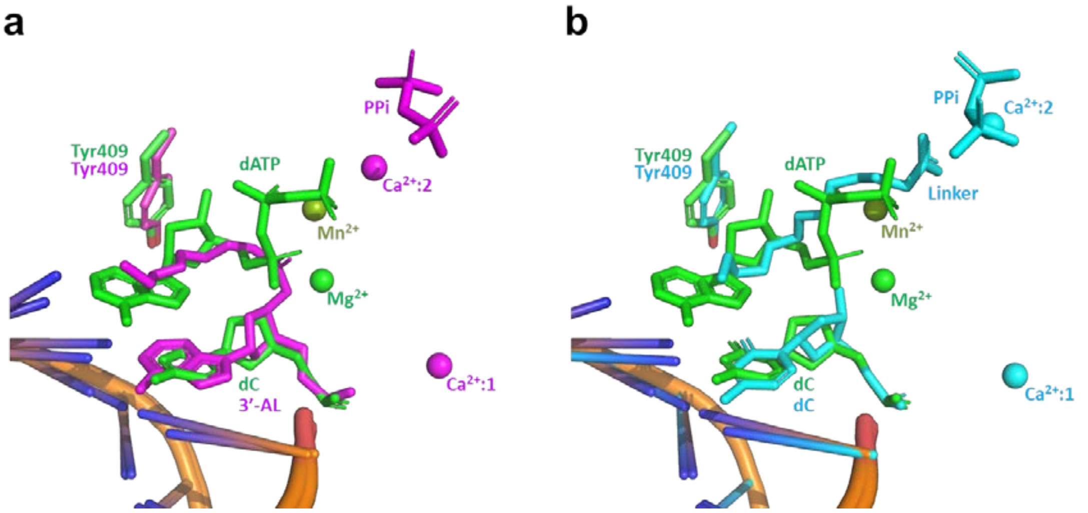

Supplementary Fig. 6. Comparison of the nucleotides in the active site. Alignment of the P/T duplex DNA and the incorporated nucleotides of the solved 9°N-I/DNA/3'-AL (**a**) and 9°N-I/DNA/3'-CL (**b**) structures with the structure of the 9°N/DNA/dATP ternary complex (PDB ID: 5OMV)[19] with a dC at its primer terminus. In (**a**), the 3'-ester-linker moiety of the incorporated 3'-AL (magenta) overlaps with the incoming dATP (green) in the active site of 9°N-I. In (**b**), the 3'-ester-linker moiety of the cleaved 3'-CL (cyan) overlaps with the incoming dATP (green) in the active site of 9°N-I.

a

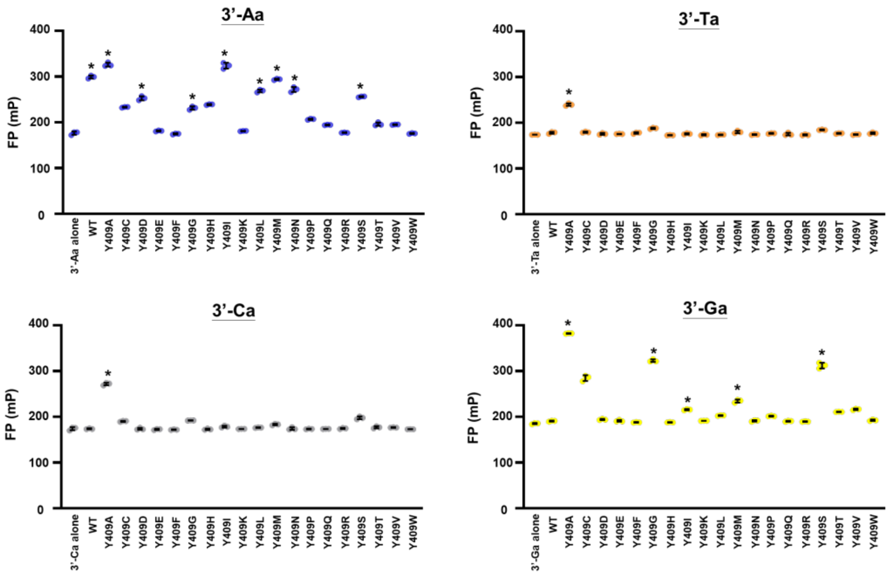

b

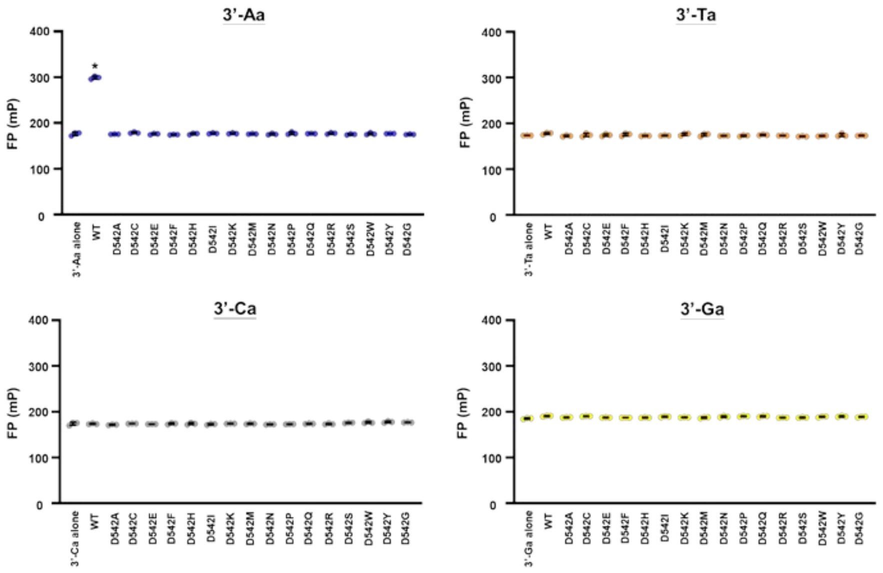

Supplementary Fig. 7. Effect of 9°N-I mutants on primer extension with 3’-Na. Extension of primer (Oligo_P#1, 1 µM) annealing to the respective template (Oligo_T#15-18, 1 µM) with 0.5 µM 3’-Na (3’-Aa, 3’-Ta, 3’-Ca and 3’-Ga) using 1 µM 9°N-I and its mutants of Y409 (**a**) and D542 (**b**) in the presence of 2 mM $MnCl_2$ was performed at 60°C for 60 min. Fluorescence polarization was measured under excitation at 535 nm and emission at 585 nm. The experiments were performed twice with three repeats (n=3) of same sample for each experiment. $^*p < 0.001$.

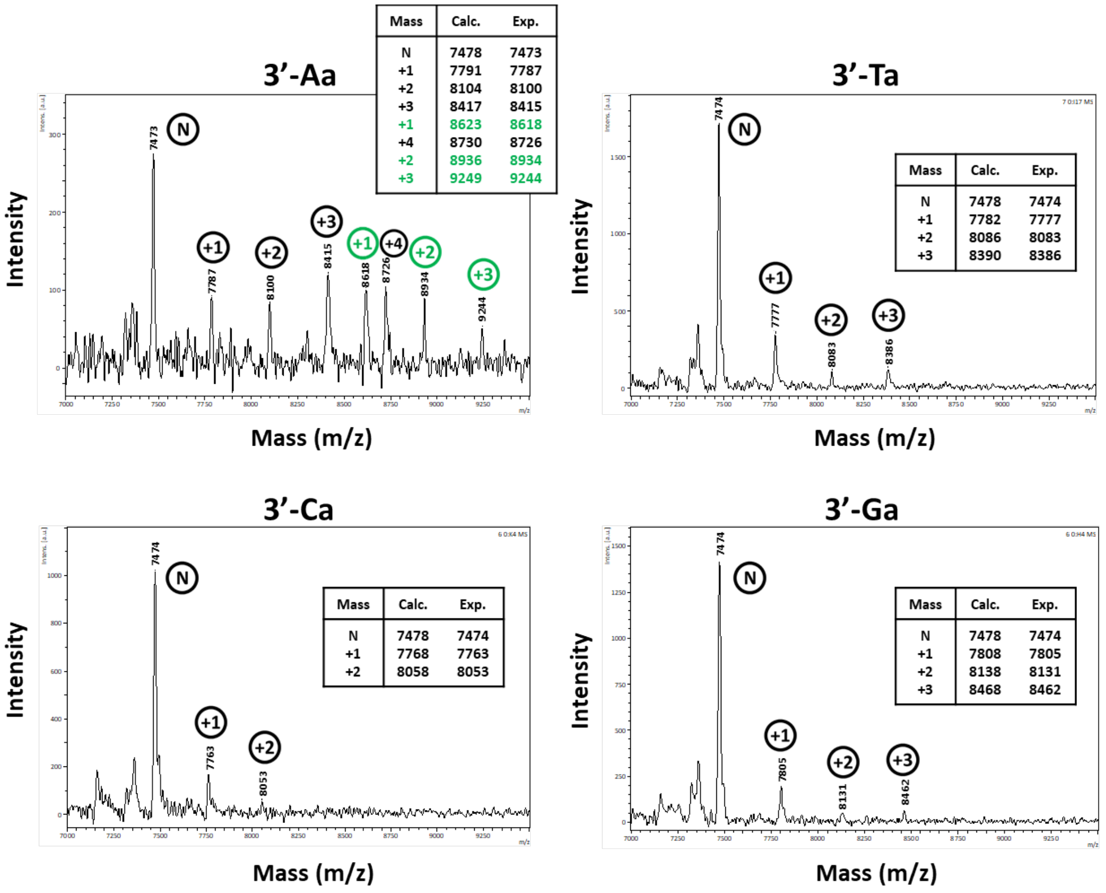

Supplementary Fig. 8. MS analysis of the primer extension with multiple 3’-Na nucleotides by 9°N-I. Extension of primer (Oligo_P#1, 0.3 µM) annealing to the respective template (Oligo_T#15-18, 0.3 µM) with 40 µM 3’-Na (3’-Aa, 3’-Ta, 3’-Ca or 3’-Ga) using 0.4 µM 9°N-I in the presence of 2 mM $MnCl_2$ was performed at 60°C for 60 min and analyzed by MALDI-TOF/TOF mass spectrometry. The translocated products of multiple bases (+1, +2 and +3) are colored in black whereas the catalytic intermediates in green. The experimental (Exp.) and calculated (Calc.) mass-to-charge ratio (m/z) of the extension peaks with the charge (z) of 1 are listed.

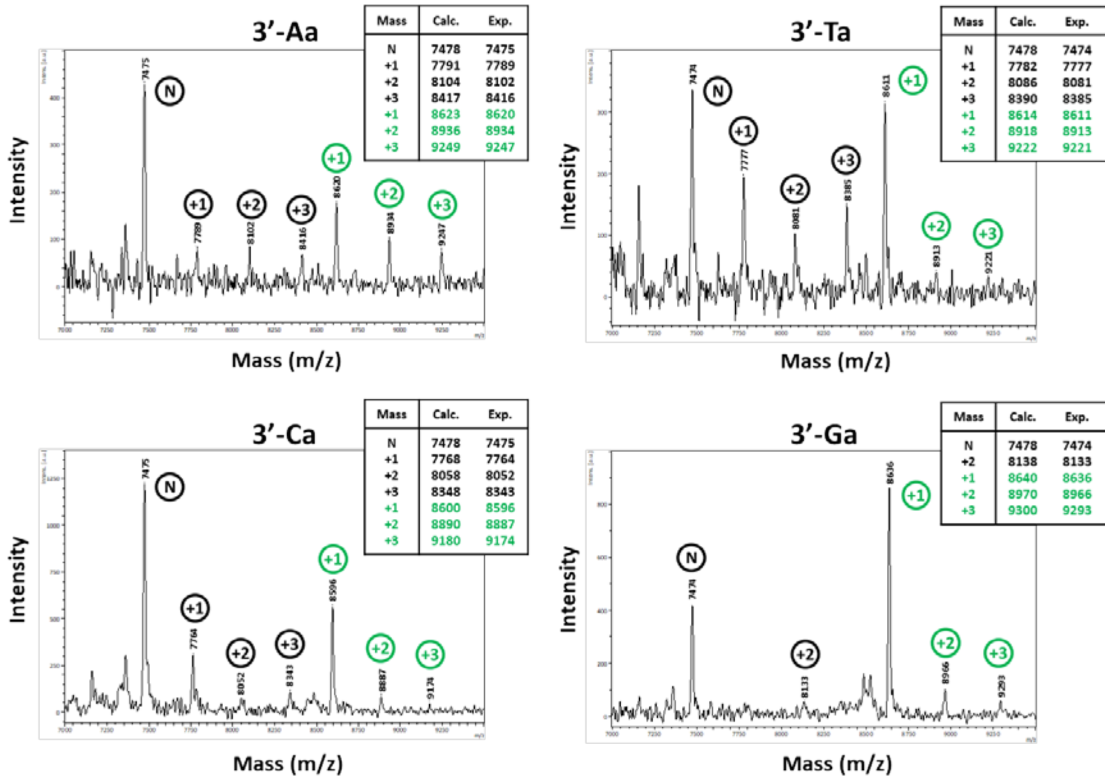

Supplementary Fig. 9. MS analysis of the primer extension with multiple 3’-Na nucleotides by Y409A under the same conditions as described in **Supplementary Fig. 8**.

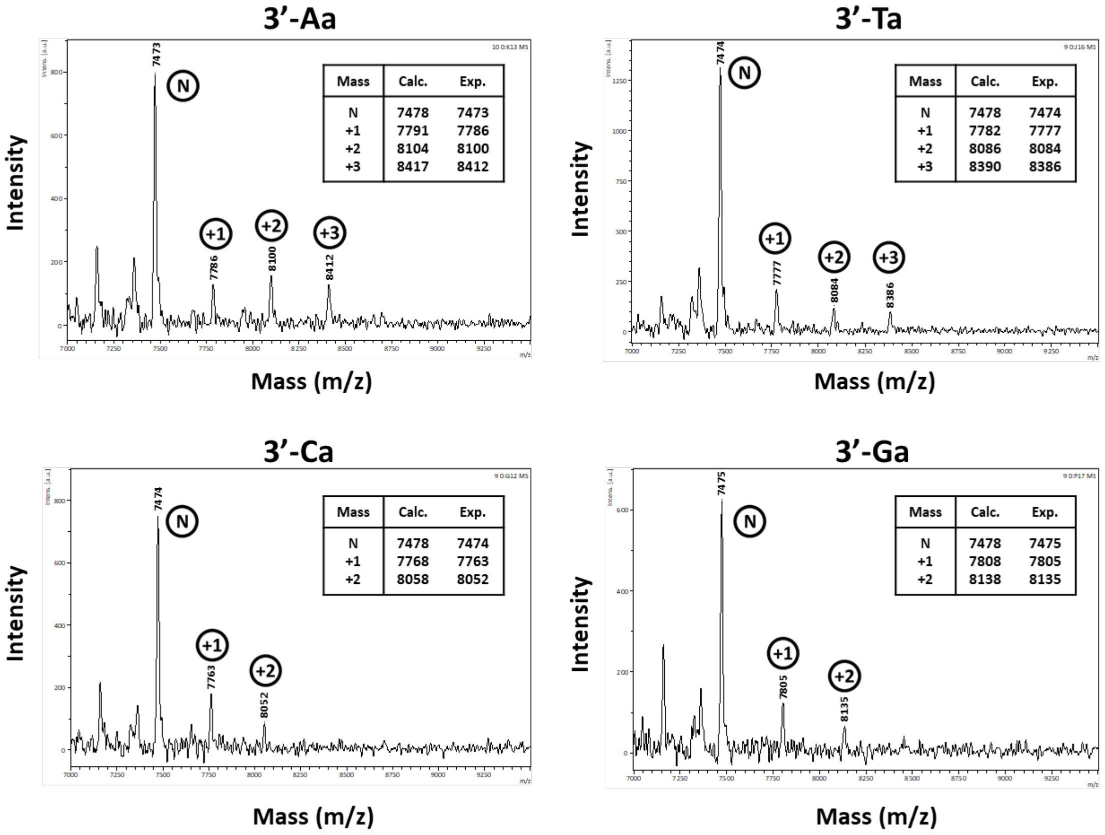

Supplementary Fig. 10. MS analysis of the primer extension with multiple 3’-Na nucleotides by D542E under the same conditions as described in **Supplementary Fig. 8**.

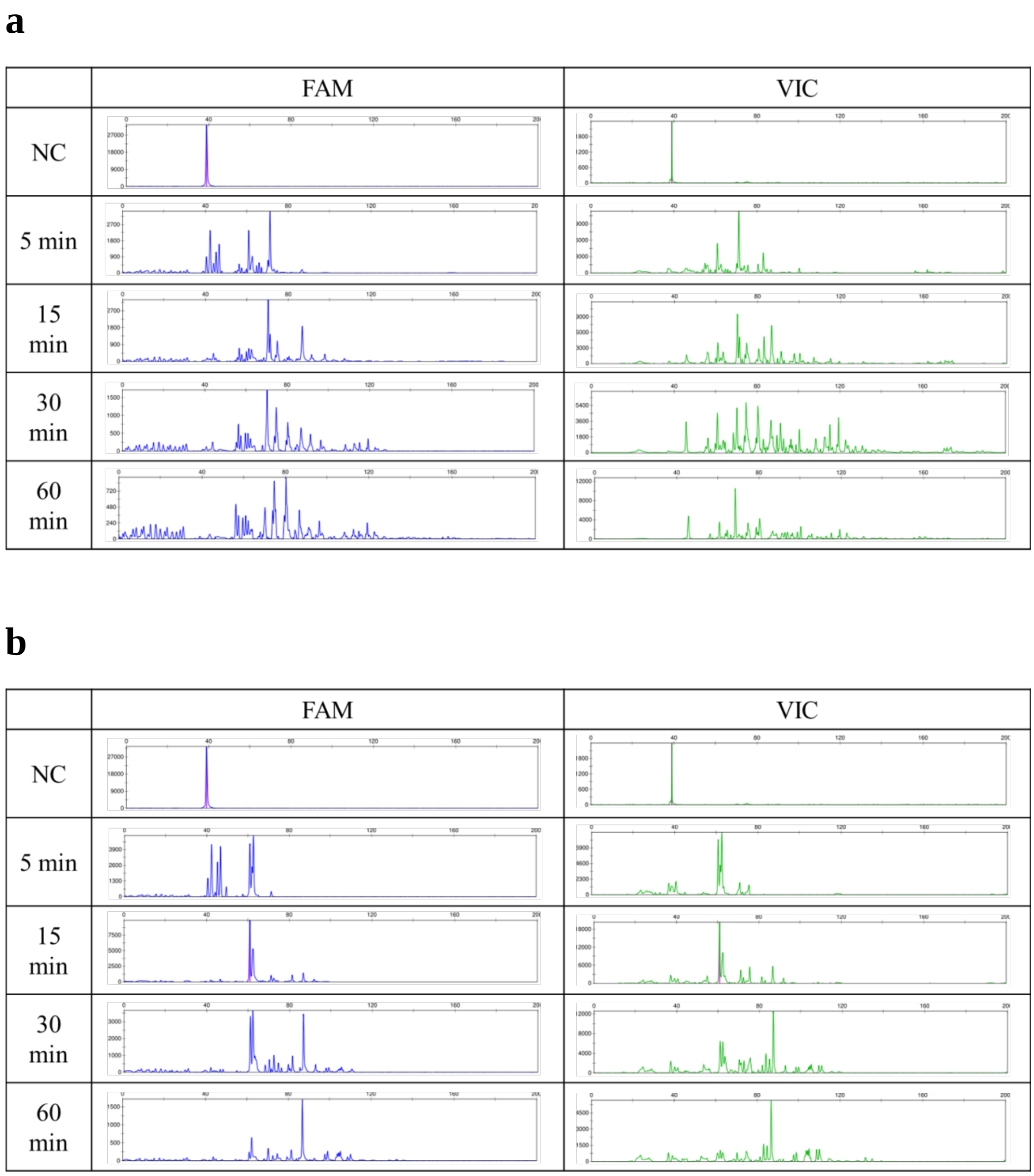

Supplementary Fig. 11. Time-dependent DNA elongation of 9°N-I and KODexo^{-} using 3’-Na. Capillary electropherograms of (**a**) 9°N-I and (**b**) KOD^{exo-} for the extension of 10 nM DNA primer by 80 μM 3’-Na at 60°C for 5, 15, 30 and 60 min. The FAM fluorescence peaks (left panel) include both the translocated products (5’-FAM-labeled oligonucleotides) and the catalytic intermediates (both 5’-FAM- and 3’-ATTO532-labeled oligonucleotides), while the VIC fluorescence peaks (right panel) represent only the catalytic intermediates.

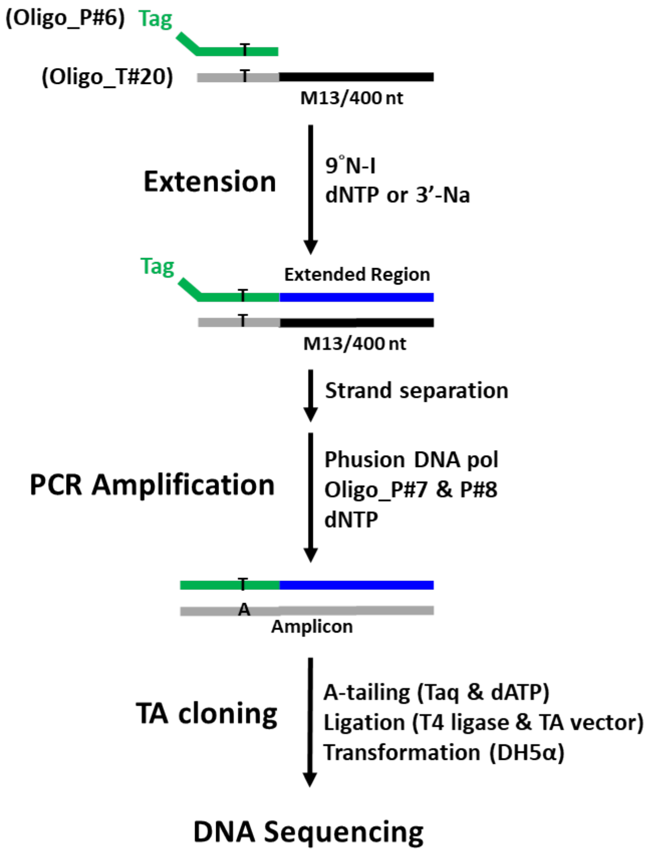

Supplementary Fig. 12. Schematic representation of the extension and amplification process used to evaluate the error rate of 3'-Na incorporation. M13 DNA template is shown in black, and the extended 3'-Na is shown in blue. The P/T duplex (Oligo_P#6 & T#20) contains a T–T mismatch, which produces a T to A transversion in the cDNA strand as a watermark for the differentiation (see **Methods**).

Supplementary Table 1. Summary of oligonucleotides used in this study.[a]

No.	Sequence (5' to 3')	Experiment
Oligo_P#1	CCCTCGCAGCCGTCCAACCAACTCA	MS of 3'-NL
Oligo_T#1	TTTGTTCTCC**T**TGAGTTGGTTGGACGGCTGCGAGGG	MS of 3'-AL<br>MS of 3'-Aa (N+1)
Oligo_T#2	TTTGTTCTCC**A**TGAGTTGGTTGGACGGCTGCGAGGG	MS of 3'-TL
Oligo_T#3	TTTGTTCTCC**G**TGAGTTGGTTGGACGGCTGCGAGGG	MS of 3'-CL
Oligo_T#4	TTTGTTCTGG**C**TGAGTTGGTTGGACGGCTGCGAGGG	MS of 3'-GL
Oligo_P#2	GCGGACTGCTTACC	Crystallization
Oligo_T#5	AC**T**GGTAAGCAGTCCGCG	Crystallization of dATP and 3'-AL
Oligo_T#6	AC**A**GGTAAGCAGTCCGCG	Crystallization of dTTP
Oligo_T#7	AC**G**GGTAAGCAGTCCGCG	Crystallization of dCTP and 3'-CL
Oligo_T#8	GA**C**GGTAAGCAGTCCGCG	Crystallization of dGTP
Oligo_P#3	FAM-AGTGAATTCGAGCTCGGTACCCGGGGATCCTCT<br>AGAGTCGACCTGCAGGC	Kinetics of dNTP and 3'-NL
Oligo_T#9	TTGCTCGTTTGCTGGG**T**GCCTGCAGGTCGACTCTAG<br>AGGATCCCCGGGTACCGAGCTCGAATTCACT	Kinetics of dATP and 3'-AL
Oligo_T#10	TTGCTCGTTTGCTAAA**G**GCCTGCAGGTCGACTCTAGA<br>GGATCCCCGGGTACCGAGCTCGAATTCACT	Kinetics of dCTP and 3'-CL
Oligo_T#11	TTGCTCGTTTGCTGGG**A**GCCTGCAGGTCGACTCTAGA<br>GGATCCCCGGGTACCGAGCTCGAATTCACT	Kinetics of dTTP and 3'-TL
Oligo_T#12	TTGCTCGTTTGCTGGG**C**GCCTGCAGGTCGACTCTAGA<br>GGATCCCCGGGTACCGAGCTCGAATTCACT	Kinetics of dGTP and 3'-GL
Oligo_P#4	CCCTCGCAGCCGTCCAACCAACTCC[x]	Dideoxy primer for FP of 3'-Na
Oligo_T#13	TTTGTTCG**TTT**GGAGTTGGTTGGACGGCTGCGAGGG	Template annealing to dideoxy primer for FP of 3'-Aa
Oligo_T#14	TTTGTTCGC**TT**TGAGTTGGTTGGACGGCTGCGAGGG	MS of 3'-Aa (N+2)
Oligo_T#15	TTTGTTCG**TTT**TGAGTTGGTTGGACGGCTGCGAGGG	MS and FP of 3'-Aa (N+3)
Oligo_T#16	TTTGTTCT**AAA**TGAGTTGGTTGGACGGCTGCGAGGG	MS and FP of 3'-Ta
Oligo_T#17	TTTGTTCT**GGG**TGAGTTGGTTGGACGGCTGCGAGGG	MS and FP of 3'-Ca
Oligo_T#18	TTTGTTCT**CCC**TGAGTTGGTTGGACGGCTGCGAGGG	MS and FP of 3'-Ga
Oligo_P#5	FAM-CGAGCACGTATAACGTGCTTTCCTCGTTGGA<br>ATCAGAGCGGGAGCTAAAC	DNA elongation
Oligo_T#19	AAATATTGTCTGTGCCACGTATTCTTACGCTTTCAGGTCA<br>GAAGGGTTCTATCTCTGTTGGCCAGAATGTCCCTTTTATT	DNA elongation

	ACTGGTCGTGTGACTGGTGAATCTGCCAATGTAAATAATCCATTTCAGACGATTGAGCGTCAAAATGTAGGTATTTCCATGAGCGTTTTTCCTGTTGCAATGGCTGGCGGTAATATTGTTCTGGATATTACCAGCAAGGCCGATAGTTTGAGTTCTTCTACTCAGGCAAGTGATGTTATTACTAATCAAAGAAGTATTGCTACAACGGTTAATTTGCGTGATGGACAGACTCTTTTACTCGGTGGCCTCACTGATTATAAAAACACTTCTCAAGATTCTGGCGTACCGTTCCTGTCTAAAATCCCTTTAATCGGCCTCCTGTTTAGCTCCCGCTCTGATTCCAACGAGGAAAGCACGTTATACGTGCTCG	
Oligo_P#6	CTTTTAAGAACCGGACGAACCGAGCACGT**T**TAACGTGCTTTCCTCG	Amplification for sequencing<br>"**T**": watermark
Oligo_T#20	AAATATTGTCTGTGCCACGTATTCTTACGCTTTCAGGTCAGAAGGGTTCTATCTCTGTTGGCCAGAATGTCCCTTTTATTACTGGTCGTGTGACTGGTGAATCTGCCAATGTAAATAATCCATTTCAGACGATTGAGCGTCAAAATGTAGGTATTTCCATGAGCGTTTTTCCTGTTGCAATGGCTGGCGGTAATATTGTTCTGGATATTACCAGCAAGGCCGATAGTTTGAGTTCTTCTACTCAGGCAAGTGATGTTATTACTAATCAAAGAAGTATTGCTACAACGGTTAATTTGCGTGATGGACAGACTCTTTTACTCGGTGGCCTCACTGATTATAAAAACACTTCTCAAGATTCTGGCGTACCGTTCCTGTCTAAAATCCCTTTAATCGGCCTCCTCGAGGAAAGCACGTTA**T**ACGTGCTCG	Amplification for sequencing<br>"**T**": watermark
Oligo_P#7	CTTTTAAGAACCGGACGAACCGAG	Amplification for sequencing
Oligo_P#8	CTGGATATTACCAGCAAGGCCGAT	Amplification for sequencing

[a]The underlined sequence is used for primer annealing, and the bolded base is used for specific nucleotide incorporation.

Supplementary Table 2. Summary of emission peaks obtained by capillary electrophoresis after DNA elongation.[a]

No.	6-FAM			VIC			Size Shift[b]
	Size	Height	Area	Size	Height	Area	
**1**	160.85	279	1113	163.41	82	470	**+2.56**
**2**	163.17	217	1052	165.72	75	323	**+2.55**
**3**	164.02	62	271	166.45	97	546	**+2.43**
**4**	172.88	222	893	175.29	64	298	**+2.41**
**5**	176.02	185	836	178.31	57	250	**+2.29**
**6**	177.11	193	803	179.40	114	627	**+2.29**
**7**	180.36	228	916	182.79	83	537	**+2.43**
**8**	181.45	211	796	183.63	78	571	**+2.18**
**9**	184.60	317	1407	187.02	94	514	**+2.42**
**10**	197.12	217	836	199.4	76	538	**+2.28**
**11**	205.57	207	884	207.83	91	689	**+2.26**
**12**	229.46	162	648	231.48	84	417	**+2.02**
**13**	235.74	128	597	237.63	75	419	**+1.89**
**14**	238.70	67	280	240.61	51	164	**+1.91**

[a]The fragments from the DNA elongation (see **Methods**) was analyzed by software, in which the fluorescently labeled fragments (6-FAM and ATTO532) were separated and sized. The analysis software uses the size standard in each reaction to create a standard curve for each sample, and then determines the relative size, height and area of each dye-labeled fragment.

[b]Size shift means the difference of size for the selected peaks between 6-FAM and VIC (the fluorescence of ATTO532). Note that there are at least four groups of size shifts obtained from the software analysis that may indicate the differentiation between 3'-Aa, 3'-Ta, 3'-Ca and 3'-Ga, including +2.55 to +2.56 (No. 1 and 2), +2.41 to +2.43 (No. 3, 4, 7 and 9), +2.26 to +2.29 (No. 5, 6, 10 and 11) and +1.89 to +1.91 (No. 13 and 14).
